# Nanoribbon Biosensor in the Detection of miRNAs Associated with Colorectal Cancer

**DOI:** 10.3390/mi12121581

**Published:** 2021-12-18

**Authors:** Yuri D. Ivanov, Kristina V. Goldaeva, Kristina A. Malsagova, Tatyana O. Pleshakova, Rafael A. Galiullin, Vladimir P. Popov, Nikolay E. Kushlinskii, Alexander A. Alferov, Dmitry V. Enikeev, Natalia V. Potoldykova, Alexander I. Archakov

**Affiliations:** 1 Laboratory of Nanobiotechnology, Institute of Biomedical Chemistry, 119121 Moscow, Russia; goldaeva_1996@mail.ru (K.V.G.); kristina.malsagova86@gmail.com (K.A.M.); t.pleshakova1@gmail.com (T.O.P.); rafael.anvarovich@gmail.com (R.A.G.); alexander.archakov@ibmc.msk.ru (A.I.A.); 2 Rzhanov Institute of Semiconductor Physics, Siberian Branch of Russian Academy of Sciences, 630090 Novosibirsk, Russia; popov@isp.nsc.ru; 3 N.N. Blokhin National Medical Research Center of Oncology, 115478 Moscow, Russia; kne3108@gmail.com (N.E.K.); biochimia@yandex.ru (A.A.A.); 4 Institute of Urology and Reproductive Health, Sechenov University, 119992 Moscow, Russia; enikeev_dv@mail.ru (D.V.E.); potoldykovanv@gmail.com (N.V.P.)

**Keywords:** miRNA, silicon-on-insulator, nanoribbon biosensor, cancer, colorectal cancer, nanoribbon

## Abstract

A nanoribbon biosensor (NRBS) was developed to register synthetic DNAs that simulate and are analogous to miRNA-17-3p associated with colorectal cancer. Using this nanoribbon biosensor, the ability to detect miRNA-17-3p in the blood plasma of a patient diagnosed with colorectal cancer has been demonstrated. The sensing element of the NRBS was a nanochip based on a silicon-on-insulator (SOI) nanostructure. The nanochip included an array of 10 nanoribbons and was designed with the implementation of top-down technology. For biospecific recognition of miRNA-17-3p, the nanochip was modified with DNA probes specific for miRNA-17-3p. The performance of the nanochip was preliminarily tested on model DNA oligonucleotides, which are synthetic analogues of miRNA-17-3p, and a detection limit of ~10^−17^ M was achieved. The results of this work can be used in the development of serological diagnostic systems for early detection of colorectal cancer.

## 1. Introduction

Colorectal cancer is a polyetiological disease and might be caused by a number of factors, including genetic and environmental factors. Colorectal cancer is the second most common cancer-related mortality in the United States after lung cancer and is among the top three most common types of human cancer [1]. An essential point in the prevention of this disease is early diagnosis. Since the early 2000s, there has been a significant decrease in the incidence of colorectal cancer due to both its early detection and effective therapy [1]. Among these disease diagnostics, colonoscopic screening [1] and fecal occult blood tests [2] should be noted. Among the serological methods, ELISA methods using markers CEA, CA 19-9, and CA 242 [3,4] should be noted; however, these markers are not specific.

Recently, studies on using microRNAs (miRNAs) as specific markers of colorectal cancer pathogenesis [5,6,7] have appeared. Thus, these markers can be used as important targets in blood to be detected by biosensors. RNAs, whose concentration increases in blood, is convenient for use as a marker. These miRNAs include miRNA-17-3p [8], which is responsible for the regulation of cell proliferation, apoptosis, and the cell cycle [9].

Therefore, for the early stage of the disease, the detection of miRNAs in the blood at a low concentration of C <10^−14^ M is essential [10]. Serological determination of miRNA is more preferable because the procedure for preparing biomaterial for clinical research in this case is less painful compared to biopsy, which is the gold standard in cancer diagnostics [11]. On the other hand, the implementation of such methods for quantitative determination of nucleic acids such as, for example, Northern blotting, sequencing of the next generation, isothermal amplification, and quantitative polymerase chain reaction (qPCR) for early diagnosis of cancer are limited due to their lack of concentration sensitivity, multi-step protocols, and high cost [12]. Table 1 shows the concentration sensitivity of these methods. It is also important to mention that, due to the high homology of miRNA sequences [13] and the extremely small sizes of miRNA molecules themselves (length ~6–7 nm and cross-section ~2.5 nm) [14], they are extremely difficult to detect without carrying out an additional stage amplification [12].

Recently, highly sensitive biosensors operating in the field of subfemtomolar concentrations include very promising devices based on nanostructures—nanowire biosensors [23,24]. Among the nanowire biosensors, the ones based on silicon-on-insulator (SOI) structure nanochips designed with the top-down technology should be noted [25]. This technology was employed in our work for the development of nanoribbon nanochips based on field-effect transistors with n-type conductivity. These nanochips were used in a biosensor to demonstrate the detection of miRNA-17-3p associated with colorectal cancer in human blood plasma. For this, the surface of the nanochips was modified with an o-DNA probe complementary to the DNA sequence (sDNA), which is a synthetic analogue of miRNA-17-3p, associated with colorectal cancer. Thus, the study was aimed at determining the possibility of detecting miRNA-17-3p associated with colorectal cancer in biological samples using a nanoribbon biosensor. It was shown that such nanochips made it possible to register complementary sDNA, attaining a 1.1 × 10^−17^ M detection limit, and, above all, to detect an increased level of miRNA-17-3p in the blood plasma of a patient diagnosed with colorectal cancer. The advantages of the developed nanoribbon biosensor are high concentration sensitivity, as well as the possibility of detecting the studied biomarkers in biological fluid in real time without using labels. This may lay the groundwork for building highly sensitive diagnostic systems that allow detecting diseases at an early stage of their development.

## 2. Materials and Methods

### 2.1. Chemicals

Ethanol (C_2_H_5_OH, 96%) (Reakhim, Russia), isopropanol purified to 99.9% (C_3_H_8_O) (Acros Organics, Geel, Belgium), hydrofluoric acid (HF), cross-linker 3,3′-dithiobis (sulfosuccinimidyl propionate) (DTSSP) (Thermo Fischer Scientific, Waltham, MA, USA), 3-aminopropyltriethoxysilane (APTES) (Sigma-Aldrich, St. Louis, MO, USA). Auxiliary reagents: deionized water (purified with a Simplicity UV system, Millipore, Molsheim, France), potassium phosphate monobasic (KH_2_PO_4_, Sigma-Aldrich, St. Louis, MO, USA).

### 2.2. Oligonucleotides

An oligonucleotide of the following composition: CTACAAGTGCCTTCACTGCAGT, which is a DNA analog of an RNA sequence complementary to miRNA-17-3p associated with colorectal cancer, was chosen as a probe [26]. For covalent immobilization, this sequence was modified with linker type NH_2_-TTTTTTTTTT. The resulting o-DNA sequence was NH_2_-TTTTTTTTTTCTACAAGTGCCTTCACTGCAGT. The o-DNA probes were purchased from Evrogen (Moscow, Russia). A 1 µm solutions of o-DNA probes in 50 mM potassium phosphate buffer (PPB) were prepared from a stock 100 µm solution. To test the functionality of the probe oligonucleotide used, after its immobilization on the surface of the nanochip, the target sDNA was also used with the following sequence: ACTGCAGTGAAGGCACTTGTAG, which corresponded to miRNA-17-3p. A nonspecific probe of the following composition was used as a control probe: NH_2_-TTTTTTTTTTGGTCTCTGTGTTGGGCGTCTGTCTGCCCGCATGCCTGCCTCTCTGTTGCTCTGAAGGAGGCAGGGGCTGGGCCTGCAGCTGCCTGGGCAGAGCGG; the sequence corresponded to miRNA-346, which is associated with prostate cancer [27].

### 2.3. Nanoribbon Biosensor

The biosensor nanoribbon system included a sensitive nanochip, which was the bottom of a 500 μL measuring cuvette, and the cuvette itself. The solution was stirred with a stirrer in a cuvette with a rotation frequency of 3000 rpm. The nanochip was a field-effect nanotransistor designed on the basis of a silicon-on-insulator structure. SOI structures were made with the implementation of a similar Smart Cut technology [28], but with a number of differences. It is known that the technology is based on hydrogen-induced transfer of silicon layers onto the handle plate. The differences were as follows: the boundary between the top silicon layer and the hidden oxide (BOX) was a glued surface, and the BOX itself, in contrast to the Smart Cut technology, was not subjected to hydrogen implantation. This approach to the SOI structure formation reduces the risk of defects in the Si/SiO_2_ system, thereby ensuring the stability of the structure parameters. This method is described in more detail in [29]. The key design feature for optimizing the sensor sensitivity was epitaxial source-drains raised to 1 μm with a doping level of ~10^20^ cm^−3^ at a density of states in a lightly doped channel of less than 10^11^ cm^−2^ eV^−1^.

**Figure 1:**
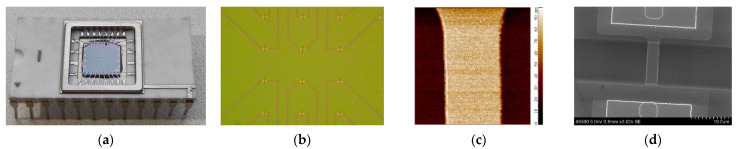
(**a**) Actual image of the nanoribbon nanochip. (**b**) Optical image of the surface of a nanochip with an array of nanoribbons; (**c**) AFM image of the nanoribbon surface obtained using a TITANIUM atomic force microscope (NT-MDT, Russia); the scan sizes are 5 × 5 µm and the resolution is 256 × 256 points. (**d**) SEM image of a nanoribbon obtained using a S-5500 scanning electron microscope (Hitachi, Japan). Nanoribbon measurements: nanoribbon thickness, t = 32 nm; nanoribbon width, w = 3 µm; nanoribbon length, l = 10 µm. The diameter of the nanochip sensor zone is ~2 mm.

On the surface of the nanochip, there were 10 nanoribbons (Figure 1a), which were elements of 10 n-type nanotransistors. Their surfaces served as virtual shutters. SOI structures had the following characteristics: buried oxide (BOX) thickness, −300 nm; the thickness of the cut-off silicon layer, −32 nm; nanoribbon thickness (t), −32 nm, nanoribbon width (w), −3 μm, nanoribbon length (l), −10 μm, and were made according to the procedure described in [30,31]. The diameter of the nanochip sensitive zone was ~2 mm. Figure 1 shows an image of a nanoribbon surface obtained using an atomic force microscope (AFM) TITANIUM (NT-MDT, Russia) (Figure 1c), as well as an image of the nanoribbon obtained using a scanning electron microscope (SEM) S-5500 (Hitachi, Ltd., Chiyoda, Tokyo, Japan) (Figure 1d).

Signal registration in digital form was carried out using a measuring unit manufactured by “Agama +” LLC (Moscow, Russia).

### 2.4. Chip Surface Modification

**Figure 2:**
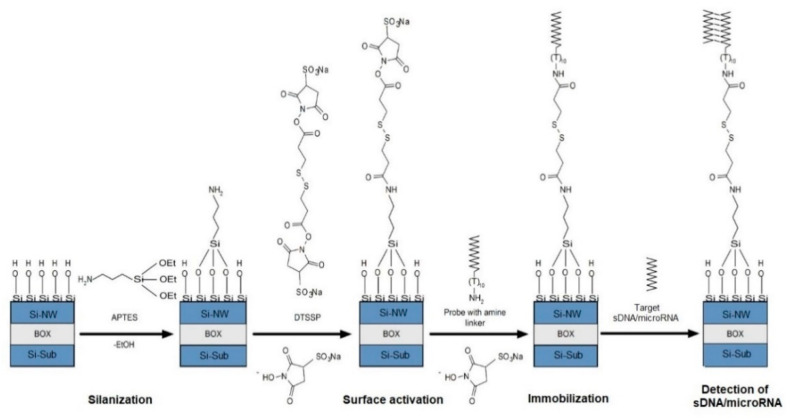
Schematic representation of the processes of chemical modification and sensitization of the nanochip surface.

The chemical modification of the nanochip included preliminary treatment of the nanochip surface followed by its silanization in APTES vapors. A schematic representation of the processes of chemical modification, as well as the subsequent sensitization of the nanochip surface, is shown in Figure 2.

An aqueous solution of isopropanol was used to remove organic and mechanical contaminants from the sensor surface of the nanochip. The next step was surface treatment to remove the natural oxide (formed during the nanochips storage) using a solution containing ethyl alcohol and HF. To form hydroxyl groups on the nanoribbon surface, the nanochip was placed in an ozonizer (UV Ozone Cleaner—ProCleaner™ Plus, Ossila Ltd., Sheffield, UK) for 60 min. Then, at room temperature in APTES vapor, the nanochip surface was silanized, similarly to the procedure described in [30,32], to produce a layer with terminal amino groups on the surface of the nanochip.

### 2.5. Covalent Immobilization of Oligonucleotide Probes

Sensitization of the surfaces of nanoribbon nanochips using o-DNA probes was carried out to ensure biospecific detection of the corresponding complementary nucleic acids of target sDNA. For this purpose, o-DNA probes were covalently immobilized on the surface of the nanochips by applying solutions of o-DNA probes (1 µm in 50 mM PPB) on the surface of nanoribbon nanochips, previously activated with a DTSSP cross-linker. For this purpose, 0.4 µg DTSSP was dissolved in a mixture containing 12 µL ethanol and 28 µL 50 mM PPB. The resulting solution was incubated in a shaker for 10 min at 4 °C and 600 rpm. After that, the resulting solution was immediately used to activate the surface of the nanochip. Then, solutions of o-DNA probes were pointwise applied to individual nanoribbons using a robotic non-contact Piezorray system (Perkin Elmer, Inc., Waltham, MA, USA). The minimum volume of the applied liquid was ~0.6 nL. The surface of the nanochip was incubated in o-DNA probes solutions at 4 °C for 30 min.

### 2.6. Preparation of Solutions of Target sDNAs in Buffer

From the stock solution (100 µm in 1 mM potassium phosphate buffer, pH 7.4) by tenfold serial dilution in the working buffer solution (1 mM potassium phosphate buffer, pH 7.4), solutions of target sDNA were obtained with a concentration of 3.3 × 10^−15^ M up to 3.3 × 10^−18^ M. At each stage of dilution, the solution was kept in a shaker at 10 °C for 30 min. Target sDNA solutions were prepared prior to measurements.

### 2.7. Electrical Measurements

A 10-channel data collection and storage system (“Agama +” LLC, Moscow, Russia) was used for electrical measurements. The substrate of the SOI structures was used as a gate. Electric current converters were employed to convert the electric current passing through the nanoribbons into voltage. Afterwards, this current was digitized by an analog-to-digital converter and graphically displayed on a PC monitor. The reading scheme is presented in more detail in [33].

In this work, the dependence of the current on the voltage at the gate, *I_ds_(V_g_)*, was monitored. The time dependencies of the current, *I_ds_(t)*, were recorded in real time at *V_g_* = 50 V and *V_ds_* = 0.2 V. A grounded Pt electrode immersed in a solution of the measuring cuvette was used to increase the stability of the system.

### 2.8. Measurements with the Nanoribbon Biosensor

To reduce the influence of the Debye shielding effect, the target sDNA was detected in a solution with a low ionic strength [34]. Potassium phosphate buffer was used as a solution with low ionic strength, as described in [35]. The detection of sDNA in the buffer was carried out according to the following scheme. First, 150 μL of 1 mM potassium phosphate buffer control solution was added to a measuring cuvette containing 300 μL of 1 mM potassium phosphate buffer to obtain the baseline. Further experiments were carried out with the introduction of the studied solutions of sDNA into the measuring cuvette in concentrations from 3.3 × 10^−15^ M up to 3.3 × 10^−18^ M in 1 mM potassium phosphate buffer. When working with miRNA samples previously isolated from blood plasma, 7 μL of miRNA solution was added to a measuring cuvette containing 100 μL of potassium phosphate buffer. The nanoribbon biosensor signals were recorded in real time.

A control experiment was carried out in a similar way to confirm the nanochip surface functionalization. The experiment was conducted using a nanochip, the surface of which was chemically activated with a DTSSP cross-linker, without subsequent covalent immobilization of o-DNA probes. In the course of the experiment, the response of the biosensor to the addition of both 150 μL of 1 mM PPB containing no sDNA (control) and the target sDNA itself with a concentration of C = 3.3 × 10^−15^ M (sample) to the measuring cuvette was recorded.

Control experiments were carried out in a similar way in order to study the effect of various interfaces on the response of the nanoribbon biosensor. In several series of experiments, either 150 μL of pure deionized water, 150 μL of pure 1 mM potassium phosphate buffer, or 150 μL of alpha-fetoprotein (a biomarker of hepatocellular carcinoma) solution, were added to the measuring cuvette of the nanobiosensor [36].

The measurement results are presented in the form of sensorgrams, i.e., the dependence of the current value on the time of the experiment is similar to [37]. The difference in signal for each nanoribbon was calculated by employing the following formula:

(1) $$\Delta I_{ds}=I_{ds}p-I_{ds}k,$$

where *I_ds_p* is the current value of the working nanowire after adding the analyzed solution and *I_ds_k* is the current value of the control nanowire after adding the analyzed solution.

To confirm the reliability of the results, the classical standard deviation function was used.

### 2.9. Plasma Samples

A blood plasma sample of patient No. 178 suffering from colorectal cancer in stage T4N1aM0 (third stage), localized in the sigmoid colon, was obtained from the National Medical Research Center of Oncology, named after N.N. Blokhin. As a control, a plasma sample of a conditionally healthy patient, No. 35, suffering from urolithiasis of a non-oncological nature, as well as a plasma sample of a patient with prostate adenocarcinoma, No. 55, were used. Control plasma samples were obtained from the Institute of Urology and Reproductive Health (Sechenov University). Plasma experiments were carried out in accordance with the order of the Ministry of Health of the Russian Federation, No. 1177н, dated 20 December 2012, and were also approved by independent ethics committees established on the basis of the organizations that provided the samples. Plasma samples were taken according to the patient examination protocol. Written informed consent was obtained from the patients to use their biological materials in the study. To ensure safety, all samples used in the experiment were decontaminated.

Fasting blood samples were taken from the cubital vein and placed into anticoagulant tubes containing 3.8% Na citrate (S-Monovett^®^, Sarstedt, Germany). The obtained samples were then centrifuged at room temperature for 6 min with a rotation frequency of 3000 rpm. An amount of 500 μL of plasma from each sample were placed in two dry test tubes. The samples were frozen and stored in a refrigerator at −80 °C.

The miRCURY™ RNA Isolation Kit - Biofluids (Exiqon A/S, Vedbaek, Denmark) was used to isolate miRNAs from blood plasma samples.

## 3. Results

### 3.1. Functionalization of the Nanochip Surface

The functionalization of the nanochip surface is a two-stage process, comprising the stages of chemical modification and sensitization.

The formation of an organosilane layer with terminal amino groups on the surface of nanoribbons took place to carry out chemical modification. Terminal amino groups were required at the stage of sensitization for covalent immobilization of o-DNA probes on the nanoribbon surface.

**Figure 3:**
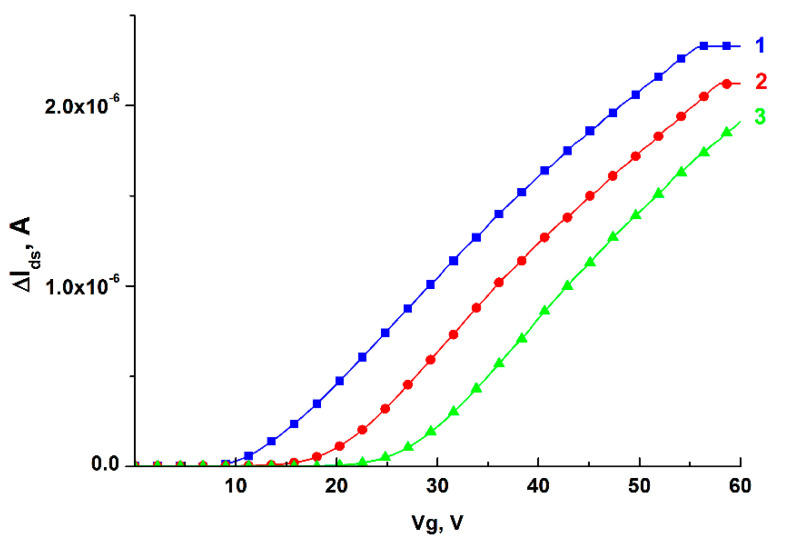
Monitoring the efficiency of functionalization of the n-type nanochip surface. The current-voltage characteristics of the nanoribbon are presented before the onset of functionalization with o-DNA probes (1), after the stage of chemical modification of the nanochip (2), and upon completion of the sensitization stage (3).

A comparative analysis of the current-voltage characteristics of the chip before and after the stage of its functionalization was carried out to control the efficiency of the functionalization of the nanochip surface. The results of measuring its characteristics for n-type nanoribbon are presented in Figure 3.

From the data presented in Figure 3, it can be inferred that, upon functionalization of the nanochip surface, the drain-gate characteristics of the functionalized nanoribbon shift to the right relative to the non-functionalized nanoribbon. The change in the nanoribbon conductivity after the stages of chemical modification and sensitization shows that functionalization of the nanochip occurs.

It should also be noted that the conductivity of silanized n-type nanowires decreases depending on pH, which corresponds to the data on the dependence of the growth of the zeta potential of SiO_2_ on a surface modified with aminopropyltriethoxysilane, presented in the work of O. Knopfmacher scientific group [38]. Negatively charged o-DNAs also lead to a decrease in conductivity due to the introduction of a negative charge [39].

**Figure 4:**
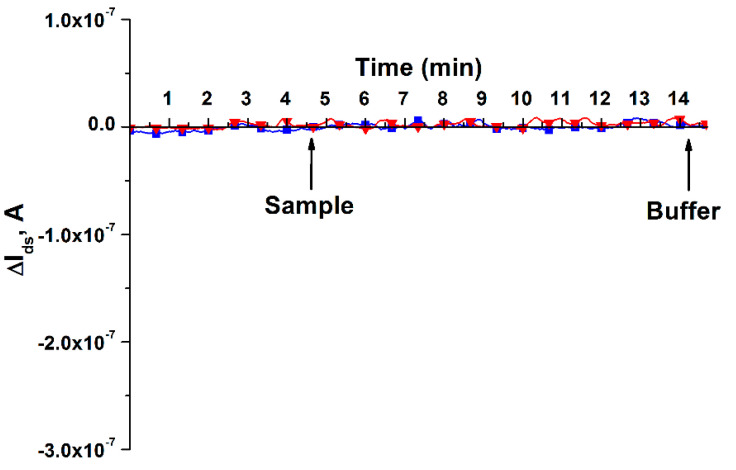
Results of detecting 1 mM PPB and target sDNA in a buffer solution using a nanochip, the surface of which was activated by DTSSP, without covalent immobilization of molecular o-DNA probes. Legend: red curve represents pure 1 mM PPB, no target sDNA; blue curve shows target sDNA with a concentration of C = 1.1 × 10^−15^ M, which is a synthetic analogue of miRNA-17-3p associated with colorectal cancer. The experiment was carried out in a working solution of 1 mM PPB (pH 7,4), *V_ds_* = 0.2 V, *V_g_* = 50 V; the solution volume in the measuring cell of the biosensor was 450 µL. Arrows indicate the moments of adding 1 mM PPB solutions or target sDNA (Sample), as well as washing potassium phosphate buffer (Buffer).

To confirm the sensitization of the nanoribbon surface, an additional experiment was also conducted using a nanochip, the surface of which was simply activated with a DTSSP cross-linker, without immobilization of o-DNA probes on the nanoribbon surface. The measurement was carried out in the *I_ds_(t)* mode. In the course of the experiment, 150 μL of pure 1 mM potassium-phosphate buffer containing no sDNA was added to the measuring cuvette of the biosensor as a control, and 150 μL of the target sDNA itself with a concentration of C = 3.3 × 10^−15^ M, which is a synthetic analogue of miRNA-17-3p associated with colorectal cancer, was used as a sample. The resulting sensorgrams are shown in Figure 4. 

The data presented in Figure 4 shows that there is no difference in the response of the nanowire biosensor, the nanoribbons of which were not immobilized by o-DNA probes, to the addition of both pure 1 mM potassium phosphate buffer and target sDNA. This indicates that there is no binding of the target sDNA, which is a synthetic analogue of miRNA-17-3p associated with colorectal cancer, to the surface of nanoribbons not immobilized by complementary o-DNA probes. This confirms the effectiveness of our method for functionalizing the surface of nanoribbons.

### 3.2. Determination of the Detection Limit Attainable with the NRBS in Buffer Solutions upon the Detection of Target sDNA

In the experiment, working nanoribbons used for biospecific detection were nanoribbons sensitized with o-DNA probes complementary to the target sDNA, which, in turn, is a synthetic analogue of miRNA-17-3p associated with colorectal cancer. The control nanoribbons used to detect non-specific binding were not sensitized with o-DNA probes.

**Figure 5:**
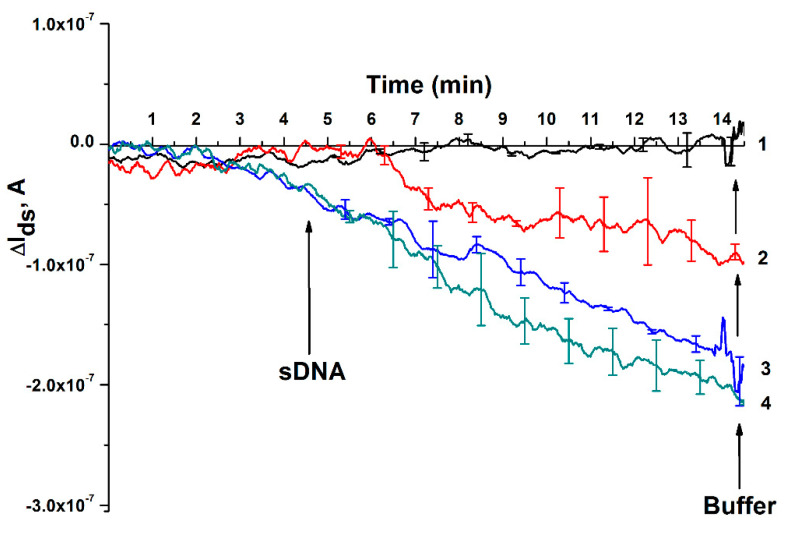
Results of target sDNA detection at various concentrations in a buffer solution employing a nanoribbon biosensor. Legend: sDNA concentration is C = 1.1 × 10^−18^ M (1); C = 1.1 × 10^−17^ M (2); C = 1.1 × 10^−16^ M (3); C = 1.1 × 10^−15^ M (4). The experiment was carried out in a working 1 mM PPB (pH 7.4), *V_ds_* = 0.2 V, *V_g_* = 50 V; solution volume was 450 μL. The number of technical repetitions is three. The arrows indicate the moments of adding the target sDNA solution and the potassium-phosphate wash buffer.

At the first stage of the experiment, solutions of target sDNA in concentrations from 3.3 × 10^−18^ M to 3.3 × 10^−15^ M were added to the measuring cuvette of the nanoribbon biosensor containing 300 µL solution of a working 1 mM potassium phosphate buffer. The reliability of the results was confirmed using the classical standard deviation function. Figure 5 shows the results of target o-DNA detection at C = 1.1 × 10^−15^ − 1.1 × 10^−18^ M concentration in a buffer solution.

Figure 5 shows that with a decrease in their concentration, a decrease in the absolute signal level was observed when adding solutions of target sDNA. Curve 1 (Figure 5) shows the signal of the nanoribbon biosensor obtained when the target sDNA solution with a concentration of 3.3 × 10^−18^ M is added to the measuring cuvette, and it can be seen from Figure 5 that, at such a concentration, the response of the biosensor signal does not change. With a tenfold increase in the concentration of target sDNA (up to 1.1 × 10^−17^ M, Figure 5, curve 2), the signal decreases after adding a 3.3 × 10^−17^ M solution of the target sDNA to the cuvette. This is due to the fact that the sDNA molecule is negatively charged as it contains phosphate groups. In the process of biospecific binding of sDNA molecules from the analyzed solution with o-DNA probes immobilized on the sensor surface, the electric charge density on the nanoribbon surface increases. This, in turn, should lead to a decrease in the electric current, *I_ds_*, passing through the nanoribbon, which is accompanied by hybridization of the captured sDNA on the nanoribbon surface. Thus, curves 1–4, presented in Figure 5, show a decrease in the response level of the signal of the nanoribbon biosensor when the concentration of the target sDNA decreases from 10^−15^ M to 10^−18^ M.

**Figure 6:**
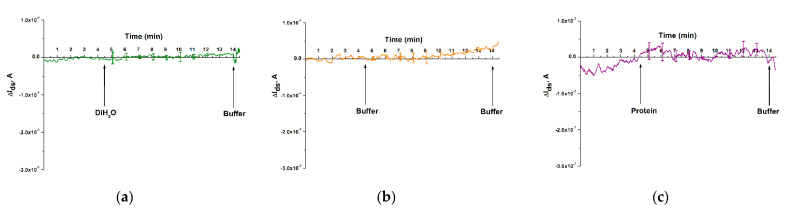
Results obtained upon addition of various test interfaces into the measuring cuvette of the nanoribbon biosensor: (**a**) pure deionized water; (**b**) pure 1 mM PPB; (**c**) alpha-fetoprotein (a protein marker of hepatocellular carcinoma). The experiment was carried out in a working solution of 1 mM PPB (pH 7.4), *V_ds_* = 0.2 V, *V_g_* = 50 V; the solution volume was 450 μL. Number of technical replicates, *n* = 3. Arrows indicate the moments of adding the test sample and washing potassium-phosphate buffer.

Further experiments to study the effect of various interfaces on the response of a nanoribbon biosensor included three series of experiments conducted: (1) adding pure deionized water to the measuring cuvette, (2) adding potassium-phosphate buffer, (3) adding a solution of the protein alpha-fetoprotein, which is a biomarker of hepatocellular carcinoma [36]. The results of these experiments are shown in Figure 6.

From the analysis of Figure 6, it can be seen that, in this series of control experiments to study the effect of various interfaces on the response of the nanoribbon biosensor, no significant change in the signal level is observed after adding neither pure deionized water and a potassium phosphate buffer not containing the target sDNA, nor alpha-fetoprotein solution to the measuring cuvette, which indicates the biospecificity of our biosensor.

Based on the analysis of the results in Figure 5 and Figure 6, it can be concluded that a biospecific interaction was observed between o-DNA probes immobilized on the surface of nanoribbons and target sDNA molecules contained in the analyzed solution. It can be inferred from Figure 2 that the minimum concentration (*C_min_*) of the target sDNA, which is a synthetic analogue of miRNA-17-3p associated with colorectal cancer, that it is possible to detected using a nanoribbon biosensor is 1.1 × 10^−17^ M.

### 3.3. Detection of miRNA Isolated from Blood Plasma using NRBS

**Figure 7:**
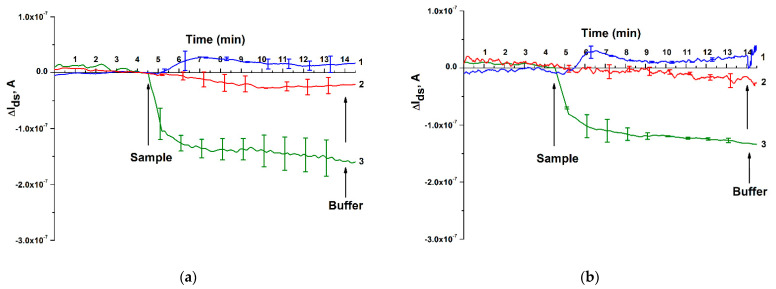
Detection of miRNA isolated from blood plasma using NRBS. (**a**) Results obtained on the first day of the study. (**b**) Results obtained on the second day of the study. Legend: curve 1—control sample of miRNA isolated from blood plasma of a conditionally healthy patient diagnosed with urolithiasis (non-oncological); curve 2—control sample of miRNA isolated from the blood plasma of a patient with adenocarcinoma of the prostate; curve 3—miRNA-17-3p sample isolated from blood plasma of a patient diagnosed with colorectal cancer. Number of technical replicates is *n* = 3. Arrows indicate the addition of miRNA solutions and 1 mM potassium phosphate wash buffer.

At this stage, the aim of the study was to determine the possibility of detecting miRNA-17-3p isolated from the blood plasma of a patient with colorectal cancer with the use of NRBS. MiRNA isolated from blood plasma of a conditionally healthy patient suffering from urolithiasis of a non-oncological nature was used as the first control (Figure 7a,b, curve 1), and miRNA isolated from blood plasma of a patient diagnosed with prostate adenocarcinoma was used as the second control (Figure 7a,b, curve 2). Experiments on the detection of samples of miRNA isolated from the blood plasma of patients were conducted on two separate days. There were three technical replicates each day. After the series of experiments at the end of the first working day, as well as at the beginning of the following working day, the sensor surface of the nanochip was rinsed with 50 mL of deionized water (72 °C) [40]. Figure 7 shows the results of detecting miRNA isolated from blood plasma in this series of experiments.

As can be inferred from Figure 7, when the miRNA-17-3p sample isolated from the blood plasma of a patient suffering from colorectal cancer was added to the NRBS measuring cuvette, there was a decrease in the signal level both on the first (Figure 7a, curve 3) and on the second (Figure 7b, curve 3) day of study. This corresponds to the expected increase in the level of negative electric charge on the nanoribbon surface caused by the capture of negatively charged miRNA, which, in turn, causes a decrease in the electric current passing through the field-effect nanotransistor. In the control experiments (Figure 7a,b, curve 1), when using siRNA isolated from the blood plasma of a conditionally healthy patient diagnosed with urolithiasis (non-oncological nature), no significant change in the signal level was observed.

Then, the possible influence of the biosensor nanochip signal on the response to the addition of miRNA isolated from the blood plasma of a patient suffering from another type of cancer, namely prostate cancer, was investigated (Figure 7a,b, curve 2). Similar to the case of a conventionally healthy patient, there was no significant decrease in the signal level in response to the addition of miRNA to the measuring cuvette of a patient with prostate cancer. Thus, Figure 7 shows that our nanoribbon biosensor responds to the addition of a sample from a patient with colorectal cancer more intensely than to control samples from a conventionally healthy patient and a patient with prostate cancer. It should be noted that the detection time of miRNA isolated from the blood plasma was only 10 min, which is significantly less than the time required for the analysis of a sample employing other molecular biological methods, described in [41,42].

The analysis of the data presented in Figure 7 also showed that multiple studies of the analyzed sample on two separate days did not reveal a remarkable change in the signal, which indicates the effectiveness of the previously developed technique for regenerating the sensor surface of a nanochip [37]. This, in turn, makes it possible to reuse the nanochip in experiments on the analysis of biological fluids.

## 4. Discussion

Herein, we have investigated the possibility of developing a nanobiosensor based on nanoribbon structures for recording sDNA, which are synthetic analogues of miRNAs associated with colorectal cancer, as well as miRNAs themselves. The nanoribbon biosensor is characterized by being a molecular detector, i.e., a device that can register single biological macromolecules and viral particles. In the case of viral particle detection, the counting mode is shown [24,43]. There are a number of theoretical works on the detection of macromolecules [29], where the ability of a molecular detector to register single macromolecules is shown. The sensitivity to single protein molecules in biological samples has not been experimentally proven [10,44], but our work has shown that, in the case of colorectal cancer, the implementation of a nanoribbon biosensor allows registering miRNAs with a sensitivity of 1.1 × 10^−17^ M. Moreover, the nanoribbon biosensor allows real-time registration, which is essential for the quick screening for oncopathologies. This method is favorably compared with the methods when molecular detectors based on atomic force microscopy (AFM) are used; despite high sensitivity for proteins (~10^−17^ M), the AFM analysis takes 3 h due to the low speed of atomic force microscopes [45]. The detection limit of ~10^−17^ M, selectivity, and the absence of labels distinguish the nanoribbon biosensor from other methods of detecting macromolecules such as surface plasmon resonance/resonant mirror (10^−13^ to 10^−12^ M) [46,47], and ELISA (10^−12^ M) [48,49]. As for molecular biological methods, e.g., polymerase chain reaction, among its disadvantages are the sensitivity to sample contamination due to the use of amplification, as well as the use of labels [49]. Therefore, nanoribbon biosensors are promising selective technologies with subfemtomolar sensitivity, allowing real-time analysis without using labels [50,51,52,53].

Therefore, this work was aimed at studying the possibility of label-free detection of miRNA in the blood of patients diagnosed with colorectal cancer. The operating principle of the nanoribbon biosensor is as follows: upon adsorption of charged target sDNA or miRNA molecules on the surface of the biospecific sensor region of the nanochip, its conductivity changes due to a change in the concentration of charge carriers on the sensor surface. The detection scheme is described in more detail in [33].

Therefore, at the beginning of the study, the sDNA calibration of the device was carried out, which was necessary for the characterization of the device. Naturally, it is desirable to calibrate the nanoribbon biosensor by miRNA; however, DNA nucleotides are known to be more stable than RNA nucleotides [54]. At the same time, it is also known that DNA sequences can be used as miRNA analogues to test the performance of nanowire devices [33,55]. Therefore, in the beginning, a nanoribbon structure based on SOI was fabricated, which would make it possible to efficiently record sDNA in buffer solutions in the subfemtomolar concentration range. Experiments in a buffer solution have shown that a nanoribbon nanochip modified with o-DNA probe molecules makes it possible to register target sDNA molecules with a concentration detection limit down to 1.1 × 10^−17^ M. According to Wu et al. [40], there is a linear relation between the logarithm of the concentration of the target sDNA captured on the nanoribbon surface and the biosensor sensitivity. In our work, the absolute value of the biosensor signal decreased with decreasing concentration in the studied range. When registering negatively charged sDNA molecules, the current *I_ds_* decreased when sDNA was added to the measuring cell, as expected for an n-type nanotransistor.

Further, the developed biosensor based on such a nanochip was tested for the possibility of using it for detecting colorectal cancer-specific miRNAs in blood. A blood sample from a stage III patient was used for this purpose. It was shown that, for this sample, there was a clear decrease in *I_ds_* after adding the sample, and this decrease was more significant than for the control samples. As control samples, a sample of a conditionally healthy patient with urolithiasis of a non-oncological nature (control 1) and a sample of a patient suffering from another type of cancer, namely prostate adenocarcinoma (control 2), were used. In the case of control 1, it was shown that practically no change in the signal of the biosensor nanochip was observed when testing this sample. In the case of control 2, the biosensor nanochip also practically did not respond to the addition of a sample prepared from the blood of this patient. This indicated good biospecificity of the nanobiosensor for the detection of miRNA-17-3p associated with colorectal cancer.

## 5. Conclusions

The study was aimed at determining the possibility of detecting miRNA-17-3p associated with colorectal cancer in biological samples using a nanoribbon biosensor. A nanochip based on SOI structures was fabricated in the form of a 10-channel array to register miRNA-17-3p associated with colorectal cancer. Previously, the efficiency of the nanochip had been tested for biospecific detection in a buffer solution of synthetic sDNA, i.e., analogues of miRNA-17-3p. The subfemtomolar (1.1 × 10^−17^ M) detection limit was attained. Further, this nanochip as a part of a biosensor was employed to register miRNA-17-3p in the blood serum of a patient diagnosed with colorectal cancer. It has been shown that the created biosensor makes it possible to register sDNA at ultralow concentrations and to detect an increased level of miRNA-17-3p in the blood in case of this disease. 

The results of the work are essential for the future development of new diagnostic devices for serologic colorectal cancer detection at an early stage. 

## Figures and Tables

**Table 1:** Basic methods for the determination of nucleic acids and their concentration sensitivity.

Biomarker	Pathology	Method	Method Sensitivity	Ref.
ncRNA	Cervical cancer	Northern blotting	10^−9^ M	[15,16]
miRNA	Breast cancer	Next-generation sequencing	10^−9^ M	[17,18]
DNA	Breast cancer	Isothermal amplification	10^−9^ M	[19,20]
miRNA	Cervical cancer	qPCR	10^−9^ M	[21,22]

## Data Availability

The data obtained throughout the experiments can be provided by Y.D.I. upon reasonable request.
